# Recipient-Related Risk Factors for Graft Failure and Death in Elderly Kidney Transplant Recipients

**DOI:** 10.1371/journal.pone.0112938

**Published:** 2014-11-12

**Authors:** Xingqiang Lai, Guodong Chen, Jiang Qiu, Changxi Wang, Lizhong Chen

**Affiliations:** Organ Transplant Center, The First Affiliated Hospital, Sun Yat-sen University, Guangzhou, China; University of Toledo, United States of America

## Abstract

**Background:**

Elderly patients with end-stage renal disease have become the fastest growing population of kidney transplant candidates in recent years. However, the risk factors associated with long-term outcomes in these patients remain unclear.

**Methods:**

We retrospectively analyzed 166 recipients aged 60 years or older who underwent primary deceased kidney transplantation between 2002 and 2013 in our center. The main outcomes included 1-, 3- and 5-year patient survival as well as overall and death-censored graft survival. The independent risk factors affecting graft and patient survival were analyzed using Cox regression analysis.

**Results:**

The 1-, 3-, 5-year death-censored graft survival rates were 93.6%, 89.4% and 83.6%, respectively. Based on the Cox multivariate analysis, panel reactive antibody (PRA)>5% [hazard ratio (HR) 4.295, 95% confidence interval (CI) 1.321–13.97], delayed graft function (HR 4.744, 95% CI 1.611–13.973) and acute rejection (HR 4.971, 95% CI 1.516–16.301) were independent risk factors for graft failure. The 1-, 3-, 5-year patient survival rates were 84.8%, 82.1% and 77.1%, respectively. Longer dialysis time (HR 1.011 for 1-month increase, 95% CI 1.002–1.020), graft loss (HR 3.501, 95% CI 1.559–7.865) and low-dose ganciclovir prophylaxis (1.5 g/d for 3 months) (HR 3.173, 95% CI 1.063–9.473) were risk factors associated with patient death.

**Conclusions:**

The five-year results show an excellent graft and patient survival in elderly kidney transplant recipients aged ≥60 years. PRA>5%, delayed graft function, and acute rejection are risk factors for graft failure, while longer duration of dialysis, graft loss and low-dose ganciclovir prophylaxis are risk factors for mortality in elderly recipients. These factors represent potential targets for interventions aimed at improving graft and patient survival in elderly recipients.

## Introduction

Kidney transplantation is considered to be the best treatment option for patients with end-stage renal disease (ESRD), regardless of their age. Currently, the mean age of patients undergoing renal transplantation has increased. This trend is observed not only in western countries such as America but also in Asian countries. Patients ≥60 years with ESRD have become the fastest growing population of wait-listed individuals and kidney transplant candidates [1], [2]. Over the last decade, both the absolute number and percent of transplants performed in patients aged ≥65 years have approximately doubled [1]. Previous studies have reported that elderly ESRD patients after kidney transplantation have lower mortality rates and improved quality of life compared with those who remain on dialysis treatment [3], [4], [5].

However, despite the known benefits of kidney transplantation over dialysis in the elderly patients, long-term outcomes of the recipients and their grafts are still limited [6]. Given the rapid increase in the number of senior renal transplant candidates combined with a growing shortage of donor kidneys, it is increasingly important to optimize the long-term outcomes in the elderly recipients. The characteristics of the elderly patients at transplantation may have an important impact on graft and patient survival. Published studies regarding the recipient factors that predict outcomes in elderly recipients are limited, especially in China. Accurately determining the possible predictors involved in graft and patient survival is crucial for improving long-term outcomes in elderly recipients.

Therefore, the aim of this study was to evaluate graft and patient survival in kidney transplant recipients aged ≥60 years and to determine the possible recipient-related risk factors associated with clinical outcome.

## Patients and Methods

This retrospective cohort study was approved by the Institutional Review Board/Ethics Committee of The First Affiliated Hospital of Sun Yat-sen University, and all aspects of the study complied with the Helsinki Declaration of 1975. The Ethics Committee of The First Affiliated Hospital of Sun Yat-sen University specifically approved that not informed consent was required because all data were going to be analyzed anonymously. All of the organs were from donation after brain death (DBD) or donation after cardiac death (DCD), and all of the organ donors had provided informed written consent. No prisoner organs were used in this study.

All patients aged 60 years or older who underwent first-time kidney transplantation from deceased donors in our center between January 2002 and June 2013 were collected. We excluded patients who had received another organ besides the kidney. The recipient characteristics included age, gender, causes of ESRD, pre-transplant comorbidities [including diabetes mellitus, hypertension, coronary artery disease (CAD)], type and time on dialysis, and panel reactive antibody (PRA) level at transplantation. The induction agents, basic maintained immunosuppressive regimens, and regimens for cytomegalovirus (CMV) prophylaxis were also recorded. After surgery, the number and frequency of adverse events including delayed graft function (DGF), acute rejection (AR) and chronic rejection (CR) at any time, leucopenia, infectious events, graft loss and patient death, malignancy and other new onset diseases were recorded. The causes of graft failure and mortality were also recorded.

Patients received IL-2 receptor antagonist (IL2RA, including basiliximab or daclizumab) or rabbit anti-thymocyte globulin (rATG) as induction agents. The maintained immunosuppressive regimens consisted of cyclosporine or tacrolimus, mycophenolate mofetil (MMF) and prednisone. From 2002 to 2006, most of the recipients received a low-dose ganciclovir (1.5 g/d for 3 months) for CMV prophylaxis, whereas patients mainly received a high-dose ganciclovir (3.0 g/d for 3 months) since 2007. Sulfamethoxazole was administered orally for 3 months for Pneumocystis jirovecii pneumonia prophylaxis.

DGF was defined as the need for dialysis in the first week after transplantation. AR was diagnosed based on clinical manifestations such as fever, oliguria, and serum creatinine elevation of >25% from the baseline value and was confirmed by a subsequent renal allograft biopsy. Biopsy-proven acute rejections (BPAR) included all acute rejections which were graded borderline or higher by Banff'97 criteria. CR was diagnosed by clinical findings with a decrease in kidney function and developing a gradual rise in serum creatinine, and was confirmed by renal allograft biopsy with histological features including thickening of the intima of arterioles and arteries, sclerosis of glomeruli, and tabular atrophy.

### Statistical analysis

All data were analyzed by SPSS for Windows Version 19.0 (SPSS, Chicago, Illinois, USA). Continuous variables are expressed as counts and percentages, and categorical variables are expressed as the means with standard deviations (mean ± SD). Actuarial graft and patient survival were calculated using Kaplan–Meier analysis. To assess variables associated with transplant outcome, univariate and multivariate Cox proportional hazards regression models were employed. The association between outcomes and all co-variables were tested separately in univariate Cox analyses. To evaluate the potential independent risk factors for transplant outcomes, all variables associated with graft loss or patient death at a *P*<0.2 level in the univariate Cox analysis were included in the final multivariate model. A *P*-value <0.05 was considered to indicate statistical significance.

## Results

### Patient characteristics

We collected data from 166 primary deceased kidney transplant recipients aged 60 or older between January 2002 and June 2013. The demographic and baseline characteristics of these elderly patients are shown in Table 1. The mean recipient age was 64.6±3.8 years, and 109 (65.7%) recipients were male. The main cause of ESRD was chronic glomerulonephritis, followed by diabetes mellitus. Hypertension was the most prevalent comorbidity in these aged recipients. The mean duration of dialysis was 18.6±22.1 months. Initial immunosuppressive induction therapy based on IL2RA was 68.1% and that based on rATG was 31.9%. At baseline, 56.6% and 43.4% of patients were on cyclosporine-based and tacrolimus-based immunosuppression, respectively; for CMV prophylaxis, 101 (60.8%) recipients received low-dose ganciclovir (1.5 g/d for 3 months), and 65 (39.2%) received high-dose ganciclovir (3.0 g/d for 3 months).

**Table 1:** Recipient baseline characteristics (N = 166).

Recipient age, yr (mean±SD)	64.6±3.8	Dialysis, n (%)	
Male recipients, n (%)	109 (65.7)	Nondialysis	46 (27.7)
Cause of ESRD, n (%)		Hemodialysis	87 (52.4)
Chronic glomerulonephritis	73 (44)	Peritoneal dialysis	33 (19.9)
Diabetes mellitus	51 (30.7)	Time on dialysis, months (mean±SD)	18.6±22.1
Hypertension	19 (11.4)	PRA>5%, n (%)	16 (9.6)
Obstructive nephropathy	7 (4.2)	IL2RA, n (%)	113 (68.1)
Polycystic kidney	6 (3.6)	rATG, n (%)	53 (31.9)
Others or unknown	10 (6)	Cyclosporine, n (%)	94 (56.6)
Comorbidities, n (%)		Tacrolimus, n (%)	72 (43.4)
Diabetes mellitus	70 (42.1)	Low-dose ganciclovir (1.5 g/d), n (%)	101 (60.8)
Hypertension	120 (72.3)	High-dose ganciclovir (3.0 g/d), n (%)	65 (39.2)
Coronary artery disease (CAD)	24 (14.5)		

### Graft and patient survival

**Figure 1:**
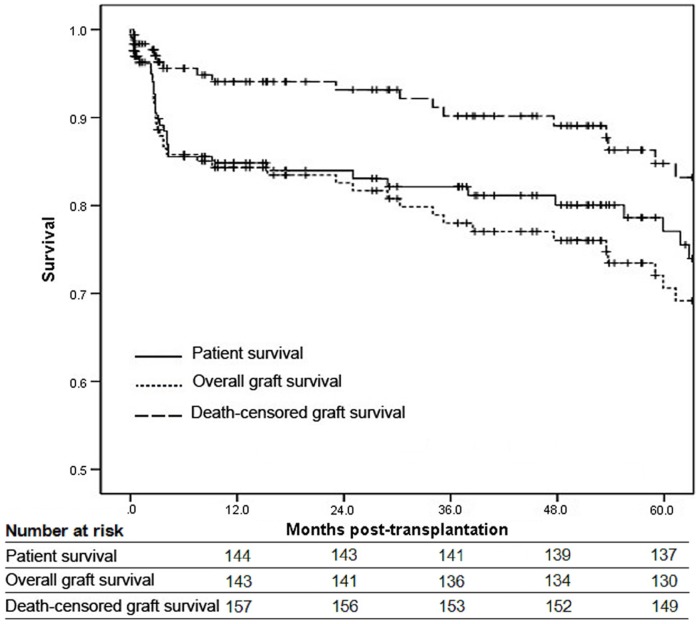
Patient survival, overall and death-censored graft survival.

The adverse events during the 5-year follow-up are shown in Table 2. The incidence of DGF, AR and chronic rejection (CR) was 9%, 16.9% and 8.3%, respectively. Infection was the most common adverse event in the elderly patients, with an incidence of 55.4%. The incidence of CMV infection was 17.5%, and most of which occurred in the first year post-transplantation. A total of 36 patients experienced graft loss and 29 patients died within 5-year follow-up. Overall and death-censored graft survival and patient survival are shown in Figure 1. The 1-, 3-, 5-year overall graft survival was 84.3%, 78% and 70.6%, respectively. However, when patient death was not considered as graft loss (death-censored), the 1-, 3-, 5-year graft survival reached 93.6%, 89.4% and 83.6%, respectively. Patient survival was 84.8% at 1 year, 82.1% at 3 year and 77.1% at 5 year. The causes of graft loss and patient death are shown in Table 3. The main causes of graft loss were patient death (52.8%) and AR (16.7%). Most of the patients died of infection (55.2%) and CAD (17.2%). In addition, among the infectious mortality, there were 11 deaths due to severe CMV disease.

**Table 2:** Adverse events in 5-year follow-up.

Adverse events	n (%)
DGF	15 (9)
AR	28 (16.9)
CR	3 (8.3)
Leukopenia	9 (5.4)
All infections	92 (55.4)
Urinary tract	7 (4.2)
Probable bacterial or other	35 (21.1)
Confirmed bacterial	64 (38.6)
CMV	29 (17.5)
BK polyoma virus	1 (0.6)
Fungal	10 (6)
Liver impairment	39 (23.5)
New onset diabetes mellitus	18 (10.8)
Congestive heart failure	8 (4.8)
Cerebrovascular accident	4 (2.4)
Malignancy	9 (5.4)
Graft loss	36 (21.7)
Death	29 (17.5)

**Table 3:** Causes of graft loss and mortality (5 years).

Graft loss (N = 36)	n (%)	Mortality (N = 29)	n (%)
Patient death	19 (52.8)	Infection	16 (55.2)
AR	6 (16.7)	CAD	5 (17.2)
CR	3 (8.3)	Cerebrovascular accident	2 (6.9)
Chronic allograft nephropathy	4 (11.1)	Malignancy	3 (10.3)
Recurrence	1 (2.8)	Liver disease	1 (3.5)
ARF	1 (2.8)	Hemorrhage	1 (3.5)
PNF	1 (2.8)	Unknown	1 (3.5)
Technical failure	1 (2.8)		

ARF, acute renal failure; PNF, primary no function.

### Risk factors for graft loss

During a 5-year follow-up, there were 36 cases of graft loss in the cohort. More than half of the graft losses (52.8%) were due to patient death. Univariate analysis showed that longer dialysis time, PRA>5%, DGF and AR were risk factors for death-censored graft loss. Based on the Cox multivariate models, a PRA>5% [hazard ratio (HR) 4.295, 95% confidence interval (CI) 1.321–13.97], DGF (HR 4.744, 95% CI 1.611–13.973) and AR (HR 4.971, 95% CI 1.516–16.301) remained independent risk factors for death-censored graft loss, except for longer dialysis time (Table 4). The status of comorbidities, including diabetes mellitus, hypertension and CAD were not associated with shorter graft survival.

**Table 4:** Risk factors for death-censored graft loss (5 years).

Variables	Univariate analysis			Cox multivariate analysis		
	HR	95% CI	P value	HR	95% CI	P value
Age (1-year increase)	1.059	0.95–1.179	0.3	—	—	—
Gender (male vs female)	1.255	0.464–3.397	0.655	—	—	—
Dialysis time (1-month increase)	1.013	1.001–1.026	0.028	1.014	0.999–1.029	0.061
Diabetes mellitus	0.624	0.231–1.691	0.354	—	—	—
Hypertension	2.016	0.458–8.866	0.353	—	—	—
CAD	0.638	0.146–2.795	0.551	—	—	—
PRA>5%	10.503	4.037–27.325	<0.001	4.295	1.321–13.97	0.015
Induction (IL2RA vs rATG)	0.553	0.156–1.959	0.359	—	—	—
Cyclosporin vs tacrolimus	2.05	0.778–5.396	0.146	1.322	0.495–3.534	0.577
DGF	5.908	2.059–16.954	0.001	4.744	1.611–13.973	0.005
AR	6.782	2.612–17.609	<0.001	4.971	1.516–16.301	0.008

HR, hazard ratio; CI, confidence interval.

### Risk factors for patient death

There were 29 deaths during the 5-year follow-up. The risk factors for patient death were shown in Table 5. Univariate analysis showed that longer dialysis time, AR, graft loss and low-dose ganciclovir prophylaxis were risk factors for patient death. However, when data was analysis by final Cox multivariate model, we found that longer dialysis time (HR 1.011 for 1-month increase, 95% CI 1.002–1.020), graft loss (HR 3.501, 95% CI 1.559–7.865) and low-dose ganciclovir prophylaxis (HR 3.173, 95% CI 1.063–9.473) remained significant independent risk factors for mortality. AR, diabetes mellitus, hypertension, CAD, and DGF were not significantly associated with patient death.

**Table 5:** Risk factors for patient death (5 years).

Variables	Univariate analysis			Cox multivariate analysis		
	HR	95% CI	P value	HR	95% CI	P value
Age (1-year increase)	1.068	0.974–1.171	0.16	1.081	0.985–1.186	0.1
Gender (male vs female)	1.243	0.577–2.675	0.579	—	—	—
Dialysis time (1-month increase)	1.013	1.004–1.022	0.004	1.011	1.002–1.020	0.02
Diabetes mellitus	0.713	0.336–1.51	0.377	—	—	—
Hypertension	0.924	0.393–2.171	0.856	—	—	—
CAD	1.412	0.574–3.47	0.452	—	—	—
Induction (IL2RA vs rATG)	0.438	0.166–1.155	0.095	1.627	0.38–6.966	0.512
CsA vs FK506	1.627	0.777–3.407	0.197	1.554	0.735–3.289	0.249
DGF	2.345	0.889–6.185	0.085	2.303	0.856–6.196	0.098
AR	3.595	1.697–7.617	0.001	1.91	0.715–5.101	0.197
Graft loss	4.571	2.073–10.081	<0.001	3.501	1.559–7.865	0.002
Ganciclovir (1.5 g/d vs 3 g/d)	3.947	1.366–11.401	0.011	3.173	1.063–9.473	0.039

## Discussion

The rapid increase in elderly patients with ESRD has raised an important issue regarding the optimization of long-term outcomes in this population. Despite a higher percentage of cadaveric kidney donors being allocated to older patients, the short-term graft survival is excellent in the majority of these patients. However, longer-term graft survival in the elderly recipients is less than expected due to death with a functioning graft, which is the major cause of graft loss. Elderly renal transplant recipients are at increased risk of graft loss and death compared with younger cohorts [7], [8]. Considering the rapid growth of an aging ESRD population and the shortage of donor kidneys, identifying the possible risk factors of graft and patient survival in elderly recipients is crucial for improving their long-term outcome.

In this study, we retrospectively analyzed 166 cases of renal transplantation from deceased donors in recipients aged ≥60 years and identified the possible risk factors predicting poor clinical outcome. Consistent with previous studies, death with a functioning graft is the leading cause of graft loss in elderly patients, with a percentage of more than 50% [9]. When patient death was not considered as the cause of graft loss, the 1-, 3-, 5-year graft survival reached 93.6%, 89.4% and 83.6%, respectively. Similar to previous studies, a PRA>5% at the time of transplantation was a significant independent risk factor for graft loss in recipients aged ≥60 years. Heldal et al. [10] reported a PRA>5% as a risk factor for graft loss in patients aged ≥70 years, whereas Faravardeh et al. [11] demonstrated that a PRA>10% was a risk factor for graft failure in recipients aged ≥65 years.

In our study, we found that patients aged ≥60 years experienced a low incidence of AR episodes, which were similar to previous findings [12]. Although the incidence of AR was lower in the elderly transplant recipients, the impact of AR episodes was far more severe than in the young recipients [13]. We found that both DGF and AR were risk factors for graft loss in recipients aged ≥60 years. Heldal et al. [10] reported that DGF was an independent predictor for death-censored graft loss in the elderly recipients aged 60 years or older. Similarly, in the study by Faravardeh et al. [11], DGF and AR were risk factors for graft failure not only in younger recipients but also in elderly cohorts aged ≥65 years. In a multicenter case-control study in Spain, Moreso et al. [14] also confirmed that AR was an independent predictor of death-censored graft failure in adult renal transplant recipients. However, in another prospective multicenter study performed in Spain, the authors did not find any significant association between AR and allograft loss in neither recipients aged ≥60 years nor in younger recipients [15]. Nevertheless, AR and DGF can result in functional and structural damage to the graft, which leads to late poor graft outcomes. Therefore, a decrease in the incidence of AR or DGF may result in an improvement of late graft outcome.

The literature on the association between duration of dialysis and transplant outcome is rich and at times inconsistent. Most of the studies reported that longer duration of dialysis was associated with poorer patient and graft outcome in kidney transplant recipients [16], [17], [18], whereas some other studies didn't find any association between dialysis duration and patient or graft survival [15]. In addition, there are also some studies reported that longer time on dialysis was independent risk factor only for patient death, but not for graft loss [9], [19]. In the study by Faravardeh et al. [11], the authors reported that longer dialysis time was a risk factor for graft loss and mortality in recipients aged <50 years, but not in those aged ≥50 years. Therefore, the risk of transplant outcome associated with increased dialysis duration time may differ between various countries and populations. In this retrospective study, we demonstrated that increased time on dialysis was an independent risk factor for patient death. Since the maintenance of dialysis status may accelerate cardiovascular changes, including vascular calcification, left ventricular hypertrophy and congestive heart failure [20], longer time on dialysis may result in an increased risk of death, especially in the elderly with a higher incidence of vascular complications. In the current study, we did not find longer duration of dialysis to be a significant independent risk factor for graft failure in multivariate Cox regression model. This finding may be explained by the fact that the elderly may differ somewhat from the younger population, since the incidence of acute rejection appears to steadily reduce with increased age [1]. In addition, the mean duration of pretransplant dialysis in our cohort is 18.6 months, which is relatively short compared to previous studies. Furthermore, the relatively small sample may somewhat weaken the impact of dialysis time on graft survival.

Graft failure was a significant risk factor for mortality for patients aged ≥60 years in our study, which was confirmed in previous studies [11]. The increased death rate was partly due to the potential immediate complications of graft loss and longer-term worse survival of returning to dialysis.

CMV infection is a common problem in immunocompromised hosts and an important cause of morbidity and mortality in kidney transplant recipients [21]. Previous studies had demonstrated that CMV infection was associated with acute and chronic graft rejection [22], increased incidence of opportunistic infection [23], graft loss and decreased recipient survival [24]. Universal prophylaxis with effective antiviral agents is one of the possible approaches for prevention of CMV infection. In China, ganciclovir remains the preferred agent for prophylaxis and treatment of CMV infection due to its effectiveness and affordable price to most patients. The study by Ahmed and colleagues demonstrated that low-dose ganciclovir (1.0 g/d for 3 months) was as effective at decreasing the incidence of clinical CMV disease as high-dose ganciclovir (3.0 g/d for 3–6 months) in renal transplant recipients [25]. However, in the present study, we found that a low-dose ganciclovir (1.5 g/d for 3 months) was significantly associated with shorter patient survival in elderly recipients. This difference could be explained by the fact that the mean age of Ahmed's recipients was 46.1±2.2 years, which was much lower than those in our study. Elderly patients are thought to generate a less robust immune response due to a decreased immunogenicity with increased age [26] combined with more pre-transplant comorbidities, which may increase the overall risk of infectious death in elderly transplant recipients [27], [28]. Low-dose ganciclovir may be not as effective as high-dose ganciclovir for CMV prophylaxis in elderly recipients. Therefore, it is important to widely use a high-dose ganciclovir to decrease the incidence of CMV infection in the more susceptible population such as the elderly renal transplant recipients.

CAD was one of the most common comorbidities and causes of death in elderly recipients. Faravardeh et al. [11] reported that CAD was a risk factor for mortality in both recipients aged ≥65 years and the younger cohort aged <50 years, while Heldal et al. [10] found that CAD increased mortality in transplant recipients up to 70 years but not in older recipients. However, in our study, we did not find CAD to be a risk factor for mortality in patients aged ≥60 years at transplantation, which was consistent with Doyle's finding [29]. The lower prevalence of CAD in the elderly recipients may be a likely explanation for our finding because there were fewer patients with CAD in our cohort and this likely led to a loss of some power to detect the significance of this factor. This is not surprising, as China is known traditionally for low incidence of CAD and low plasma cholesterol levels due to the Chinese diet and lifestyle [30], [31]. Although the incidence of CAD has increased in the last 2 decades, the incidence attained is still significantly lower than that in the Western countries [32]. Furthermore, the morbidity and mortality of CAD is much lower in south China compared with in the north [30]. Since all of the patients included were from south of China, it is reasonable that the number and proportion of CAD was low in our study. Similar to previous studies, we did not find an association between diabetes and patient survival [9], [29], [33]. We also did not find hypertension as a significant risk factor for graft failure or patient death.

Our study has certain limitations inherent in its retrospective nature because data were collected from patients transplanted from 2002 to June 2013. There were certain patients missing in the follow-up (10.5% of the overall cohort), which may reduce potential adverse effects of various risk factors that would be observed over a longer-term. In addition, the relatively small sample size might have less power to detect the effect of variables that have a smaller impact on the poor clinical outcome.

In conclusion, the transplant outcomes are excellent among recipients aged 60 years or older. We found that PRA>5%, DGF and AR were risk factors for graft failure, while longer time on dialysis, failed grafts and low-dose ganciclovir prophylaxis were risk factors for mortality in recipients aged ≥60 years. These factors represent potential targets for interventions aimed at improving graft and patient survival in elderly recipients. Therefore, reducing PRA level and decreasing the incidence of DGF and AR may result in longer graft survival, while shortening the duration of dialysis may lead to longer patient survival. In addition, high-dose ganciclovir prophylaxis should be widely used in elderly recipient to decrease the incidence of CMV infection. Nevertheless, further prospective, randomized and multicenter studies are needed to confirm these findings.

## References

[1] Danovitch GM, Gill J, Bunnapradist S (2007) Immunosuppression of the elderly kidney transplant recipient. Transplantation 84: 285–291. 17700150 10.1097/01.tp.0000275423.69689.dc

[2] McCullough KP, Keith DS, Meyer KH, Stock PG, Brayman KL, et al (2009) Kidney and pancreas transplantation in the United States, 1998–2007: access for patients with diabetes and end-stage renal disease. Am J Transplant 9: 894–906. 19341414 10.1111/j.1600-6143.2009.02566.x

[3] Wolfe RA, Ashby VB, Milford EL, Ojo AO, Ettenger RE, et al (1999) Comparison of mortality in all patients on dialysis, patients on dialysis awaiting transplantation, and recipients of a first cadaveric transplant. N Engl J Med 341: 1725–1730. 10580071 10.1056/NEJM199912023412303

[4] Johnson DW, Herzig K, Purdie D, Brown AM, Rigby RJ, et al (2000) A comparison of the effects of dialysis and renal transplantation on the survival of older uremic patients. Transplantation 69: 794–799. 10755528 10.1097/00007890-200003150-00020

[5] Oniscu GC, Brown H, Forsythe JL (2004) How great is the survival advantage of transplantation over dialysis in elderly patients? Nephrol Dial Transplant 19: 945–951. 15031354 10.1093/ndt/gfh022

[6] Meier-Kriesche HU, Schold JD, Srinivas TR, Kaplan B (2004) Lack of improvement in renal allograft survival despite a marked decrease in acute rejection rates over the most recent era. Am J Transplant 4: 378–383. 14961990 10.1111/j.1600-6143.2004.00332.x

[7] Roodnat JI, Zietse R, Mulder PG, Rischen-Vos J, van Gelder T, et al (1999) The vanishing importance of age in renal transplantation. Transplantation 67: 576–580. 10071030 10.1097/00007890-199902270-00015

[8] Meier-Kriesche H, Ojo AO, Arndorfer JA, Port FK, Magee JC, et al (2001) Recipient age as an independent risk factor for chronic renal allograft failure. Transplant Proc 33: 1113–1114. 11267215 10.1016/s0041-1345(00)02452-0

[9] Cardinal H, Hebert MJ, Rahme E, Houde I, Baran D, et al (2005) Modifiable factors predicting patient survival in elderly kidney transplant recipients. Kidney Int 68: 345–351. 15954926 10.1111/j.1523-1755.2005.00410.x

[10] Heldal K, Hartmann A, Leivestad T, Svendsen MV, Foss A, et al (2009) Clinical outcomes in elderly kidney transplant recipients are related to acute rejection episodes rather than pretransplant comorbidity. Transplantation 87: 1045–1051. 19352126 10.1097/TP.0b013e31819cdddd

[11] Faravardeh A, Eickhoff M, Jackson S, Spong R, Kukla A, et al (2013) Predictors of graft failure and death in elderly kidney transplant recipients. Transplantation 96: 1089–1096. 24056622 10.1097/TP.0b013e3182a688e5

[12] Patel SJ, Knight RJ, Suki WN, Abdellatif A, Duhart BJ, et al (2011) Rabbit antithymocyte induction and dosing in deceased donor renal transplant recipients over 60 yr of age. Clin Transplant 25: E250–E256. 21231963 10.1111/j.1399-0012.2010.01393.x

[13] Meier-Kriesche HU, Srinivas TR, Kaplan B (2001) Interaction between acute rejection and recipient age on long-term renal allograft survival. Transplant Proc 33: 3425–3426. 11750467 10.1016/s0041-1345(01)02477-0

[14] Moreso F, Alonso A, Gentil MA, Gonzalez-Molina M, Capdevila L, et al (2010) Improvement in late renal allograft survival between 1990 and 2002 in Spain: results from a multicentre case-control study. Transpl Int 23: 907–913. 20230537 10.1111/j.1432-2277.2010.01075.x

[15] Morales JM, Marcen R, Del CD, Andres A, Gonzalez-Molina M, et al (2012) Risk factors for graft loss and mortality after renal transplantation according to recipient age: a prospective multicentre study. Nephrol Dial Transplant 27 Suppl 4 v39–v46. 10.1093/ndt/gfs544 PMC3526982 23258810

[16] Meier-Kriesche HU, Port FK, Ojo AO, Rudich SM, Hanson JA, et al (2000) Effect of waiting time on renal transplant outcome. Kidney Int 58: 1311–1317. 10972695 10.1046/j.1523-1755.2000.00287.x

[17] Goldfarb-Rumyantzev A, Hurdle JF, Scandling J, Wang Z, Baird B, et al (2005) Duration of end-stage renal disease and kidney transplant outcome. Nephrol Dial Transplant 20: 167–175. 15546892 10.1093/ndt/gfh541

[18] Remport A, Keszei A, Vamos EP, Novak M, Jaray J, et al (2011) Association of pre-transplant dialysis duration with outcome in kidney transplant recipients: a prevalent cohort study. Int Urol Nephrol 43: 215–224. 20058186 10.1007/s11255-009-9700-4

[19] Helantera I, Salmela K, Kyllonen L, Koskinen P, Gronhagen-Riska C, et al (2014) Pretransplant dialysis duration and risk of death after kidney transplantation in the current era. Transplantation 98: 458–464. 24646770 10.1097/TP.0000000000000085

[20] Himmelfarb J, Ikizler TA (2010) Hemodialysis. N Engl J Med 363: 1833–1845. 21047227 10.1056/NEJMra0902710

[21] Brennan DC (2001) Cytomegalovirus in renal transplantation. J Am Soc Nephrol 12: 848–855. 11274248 10.1681/ASN.V124848

[22] Cainelli F, Vento S (2002) Infections and solid organ transplant rejection: a cause-and-effect relationship? Lancet Infect Dis 2: 539–549. 12206970 10.1016/s1473-3099(02)00370-5

[23] George MJ, Snydman DR, Werner BG, Griffith J, Falagas ME, et al (1997) The independent role of cytomegalovirus as a risk factor for invasive fungal disease in orthotopic liver transplant recipients. Boston Center for Liver Transplantation CMVIG-Study Group. Cytogam, MedImmune, Inc. Gaithersburg, Maryland. Am J Med 103: 106–113. 9274893 10.1016/s0002-9343(97)80021-6

[24] De Keyzer K, Van Laecke S, Peeters P, Vanholder R (2011) Human cytomegalovirus and kidney transplantation: a clinician's update. Am J Kidney Dis 58: 118–126. 21684438 10.1053/j.ajkd.2011.04.010

[25] Ahmed J, Velarde C, Ramos M, Ismail K, Serpa J, et al (2004) Outcome of low-dose ganciclovir for cytomegalovirus disease prophylaxis in renal-transplant recipients. Transplantation 78: 1689–1692. 15591961 10.1097/01.tp.0000141364.85454.37

[26] Martins PN, Pratschke J, Pascher A, Fritsche L, Frei U, et al (2005) Age and immune response in organ transplantation. Transplantation 79: 127–132. 15665758 10.1097/01.tp.0000146258.79425.04

[27] Meier-Kriesche HU, Ojo AO, Hanson JA, Kaplan B (2001) Exponentially increased risk of infectious death in older renal transplant recipients. Kidney Int 59: 1539–1543. 11260418 10.1046/j.1523-1755.2001.0590041539.x

[28] Kauffman HM, McBride MA, Cors CS, Roza AM, Wynn JJ (2007) Early mortality rates in older kidney recipients with comorbid risk factors. Transplantation 83: 404–410. 17318072 10.1097/01.tp.0000251780.01031.81

[29] Doyle SE, Matas AJ, Gillingham K, Rosenberg ME (2000) Predicting clinical outcome in the elderly renal transplant recipient. Kidney Int 57: 2144–2150. 10792636 10.1046/j.1523-1755.2000.00066.x

[30] Tao SC, Huang ZD, Wu XG, Zhou BF, Xiao ZK, et al (1989) CHD and its risk factors in the People's Republic of China. Int J Epidemiol 18: S159–S163. 2807697

[31] Campbell TC, Parpia B, Chen J (1998) Diet, lifestyle, and the etiology of coronary artery disease: the Cornell China study. Am J Cardiol 82: 18T–21T. 10.1016/s0002-9149(98)00718-8 9860369

[32] Ueshima H, Sekikawa A, Miura K, Turin TC, Takashima N, et al (2008) Cardiovascular disease and risk factors in Asia: a selected review. Circulation 118: 2702–2709. 19106393 10.1161/CIRCULATIONAHA.108.790048 PMC3096564

[33] Fabrizii V, Winkelmayer WC, Klauser R, Kletzmayr J, Saemann MD, et al (2004) Patient and graft survival in older kidney transplant recipients: does age matter? J Am Soc Nephrol 15: 1052–1060. 15034109 10.1097/01.asn.0000120370.35927.40

